# Hazy Al_2_O_3_-FTO Nanocomposites: A Comparative Study with FTO-Based Nanocomposites Integrating ZnO and S:TiO_2_ Nanostructures

**DOI:** 10.3390/nano8060440

**Published:** 2018-06-16

**Authors:** Shan-Ting Zhang, Guy Vitrant, Etienne Pernot, Carmen Jiménez, David Muñoz-Rojas, Daniel Bellet

**Affiliations:** 1 Université Grenoble Alpes, CNRS, Grenoble INP, LMGP, F-38000 Grenoble, France; etienne.pernot@grenoble-inp.fr (E.P.); carmen.jimenez@grenoble-inp.fr (C.J.); David.Munoz-Rojas@grenoble-inp.fr (D.M.-R.); 2 Technische Universität Darmstadt, Jovanka-Bontschits-Strasse 2, 64287 Darmstadt, Germany; 3 Université Grenoble Alpes, CNRS, Grenoble INP, IMEP-LAHC, F-38000 Grenoble, France; guy.vitrant@minatec.grenoble-inp.fr

**Keywords:** FTO, nanocomposite, Al_2_O_3_, haze factor, optical scattering, TCO

## Abstract

In this study, we report the use of Al_2_O_3_ nanoparticles in combination with fluorine doped tin oxide (F:SnO_2_, aka FTO) thin films to form hazy Al_2_O_3_-FTO nanocomposites. In comparison to previously reported FTO-based nanocomposites integrating ZnO and sulfur doped TiO_2_ (S:TiO_2_) nanoparticles (i.e., ZnO-FTO and S:TiO_2_-FTO nanocomposites), the newly developed Al_2_O_3_-FTO nanocomposites show medium haze factor H_T_ of about 30%, while they exhibit the least loss in total transmittance T_tot._ In addition, Al_2_O_3_-FTO nanocomposites present a low fraction of large-sized nanoparticle agglomerates with equivalent radius r_eq_ > 1 μm; effectively 90% of the nanoparticle agglomerates show r_eq_ < 750 nm. The smaller feature size in Al_2_O_3_-FTO nanocomposites, as compared to ZnO-FTO and S:TiO_2_-FTO nanocomposites, makes them more suitable for applications that are sensitive to roughness and large-sized features. With the help of a simple optical model developed in this work, we have simulated the optical scattering by a single nanoparticle agglomerate characterized by bottom radius r_0_, top radius r_1_, and height h. It is found that r_0_ is the main factor affecting the H_T_(λ), which indicates that the haze factor of Al_2_O_3_-FTO and related FTO nanocomposites is mainly determined by the total surface coverage of all the nanoparticle agglomerates present.

## 1. Introduction

Transparent Conductive Oxides (TCOs) constitute a unique class of thin film materials that simultaneously exhibit high optical transparency as well as good electrical conductivity. These technologically important materials are widely used in the field of photovoltaics (PV) and optoelectronics [1,2]. Recently, however, the demands on TCOs extending beyond the basic transparency and conductivity is increasing, where higher performance and multiple functions compatible with scalable, cost-efficient, and friendly processes are indeed required [3]. Due to the versatile and tunable properties of nanomaterials [4,5,6,7], an interesting strategy is to combine TCO thin films with nanomaterials in order to obtain new functional properties [8,9].

In our previous work, we have proposed a simple and cost-effective two-step approach to grow highly diffusive F:SnO_2_-based nanocomposites by integrating ZnO and S:TiO_2_ nanoparticles [10,11]. The obtained ZnO-FTO and S:TiO_2_-FTO nanocomposites exhibit superior haze factor (H_T_) of more than 60%. Using these nanocomposites as electrodes in thin film solar cells, the transmitted light could undergo scattering and thus travel longer paths in the subsequent absorber layer. Consequently, the optical absorption in the active layer is expected to be higher, thus the conversion efficiency could be potentially improved [12,13,14]. However, both nanocomposites showed decreased total transmittance (T_tot_); and S:TiO_2_-FTO nanocomposites were reported to exhibit large-sized nanoparticle agglomerates. These factors may eventually jeopardize the performance of final photovoltaics devices. In this work, we report the use of a new type of nanoparticle, Al_2_O_3_ nanoparticles, to form hazy Al_2_O_3_-FTO nanocomposites. The new nanocomposites have been fully characterized and compared with previous FTO-based nanocomposites integrating ZnO and S:TiO_2_ nanoparticles. As opposed to ZnO-FTO and S:TiO_2_-FTO nanocomposites, Al_2_O_3_-FTO nanocomposites show nanoparticle agglomerates with smaller feature sizes and the least loss of optical transmission. Finally, a simple optical model is proposed in this work to simulate the optical scattering by a single nanoparticle agglomerate. By doing so, we have successfully identified the influence of the shape parameters of the agglomerates on the optical scattering and established a direct correlation between morphological and optical properties common to Al_2_O_3_-FTO and related FTO nanocomposites. Our work here presents, for the first time, a comprehensive study of the structure-property relations for such type of FTO-based nanocomposites, which provides significant guidelines for designing and controlling the properties of hazy TCO nanocomposites for versatile applications.

## 2. Materials and Methods

### 2.1. Fabrication of Al_2_O_3_-FTO Nanocomposites Using Two-Step Approach

Al_2_O_3_ nanoparticles (average particle size: 45 nm) were purchased from Alfa Aesar (Haverhill, MA, USA), weighed and dispersed in isopropanol (Sigma Aldrich, Saint Louis, MO, USA) resulting in 3 suspensions with weight concentrations of 0.5, 1, and 2.0 wt %. In the first step, the nanoparticle suspensions were ultrasonicated for 5 min and spin-coated on glass substrates (Corning 1737) using the following single-step program: 1500 rpm (velocity) and 1500 rpm/s (acceleration) for a duration of 200 s. During spin-coating, the suspension was injected in two times with 0.2 mL each time. In the second step, a thin FTO film was conformally deposited by ultrasonic spray pyrolysis on the glass substrates coated with nanoparticles, in identical conditions with those reported in the article of Zhang et al. [15]. FTO films were deposited from a methanol (VMR^TM^, Radnor, PA, USA) solution containing SnCl_4_·5H_2_O (0.16 M) and NH_4_F (0.04 M), both purchased from Sigma Aldrich. The growth temperature was 420 °C and FTO films approximately 300 nm thick were obtained. In the same deposition batch, a bare glass substrate was positioned to acquire the reference flat FTO (same thickness of ~300 nm). Figure S1 schematically illustrates the two-step process.

### 2.2. Characterization Techniques

The surface morphologies were characterized using a field-emission FEI QUANTA FEG250 scanning electron microscopy (SEM, Waltham, MA, USA) and a Digital Instrument D3100 Nanoscope atomic force microscopy (AFM, Billerica, MA, USA). Bragg-Brentano (θ-2θ) X-ray diffraction (XRD) patterns were collected with a Bruker D8 Advance Series II diffractometer (Billerica, MA, USA) in the 2θ range of 20–70°. The sheet resistance R_s_ was acquired with an in-line four-point probe (LucasLab Probe 4 apparatus, Gilroy, CA, USA). A UV-Vis-NIR spectrophotometer equipped with an integrating sphere (PerkinElmer Lambda 950, Waltham, MA, USA) was used to record the optical transmittance and absorptance.

## 3. Results and Discussion

### 3.1. Morphological Properties of Al_2_O_3_-FTO Nanocomposites

**Figure 1:**
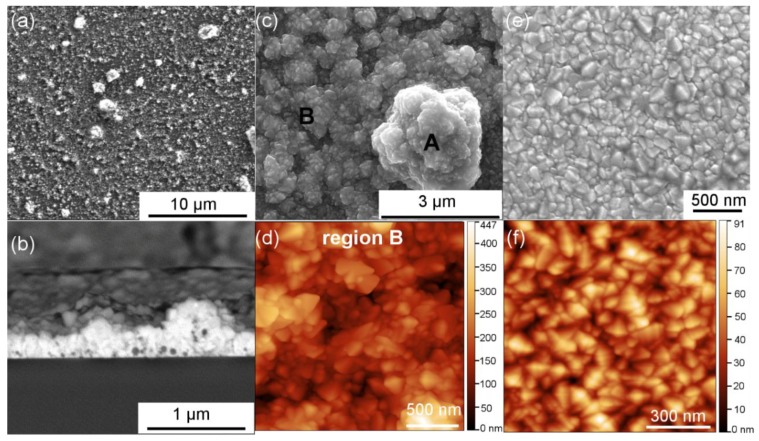
(**a**) Scanning electron microscopy (SEM) image of a 2 wt % Al_2_O_3_ nanoparticle suspension spin-coated on glass substrate; (**b**) Cross-sectional and (**c**) plane-view SEM images of a 1 wt % Al_2_O_3_-FTO nanocomposite with its region B examined in (**d**) atomic force microscopy (AFM) (2 × 2 μm^2^) image; (**e**) Plane-view SEM and (**f**) AFM (1 × 1 μm^2^) images of a reference flat FTO.

As one sees in Figure 1a, after the first step where the Al_2_O_3_ nanoparticle suspension was spin-coated on the glass substrate. The nanoparticles (appearing in bright contrast) self-assembled into agglomerates of random sizes and did not cover the entire glass surface. Then in the second step, a thin film of FTO with a thickness of ~300 nm was deposited using ultrasonic spray pyrolysis, uniformly covering the nanoparticle agglomerates, as evidenced in Figure 1b. The surface of Al_2_O_3_-FTO nanocomposites is fairly rough, as one sees in Figure 1c. Contrarily, the surface of a reference FTO (i.e., FTO film deposited directly on the bare glass substrate without any nanoparticles) shown in Figure 1e,f appears smooth and flat. The main structural and electrical properties of flat FTOs have already been investigated and discussed in previous articles [16,17,18]. Similar to S:TiO_2_-FTO nanocomposites reported in [11], Al_2_O_3_-FTO nanocomposites also show two regions: rougher region A where the FTO film was deposited on larger nanoparticle agglomerates and semi-flat region B where small nanoparticle agglomerates are present, thus resulting in higher surface roughness than a reference flat FTO (cf. compare Figure 1d,f with particular focus on the z-scale).

**Figure 2:**
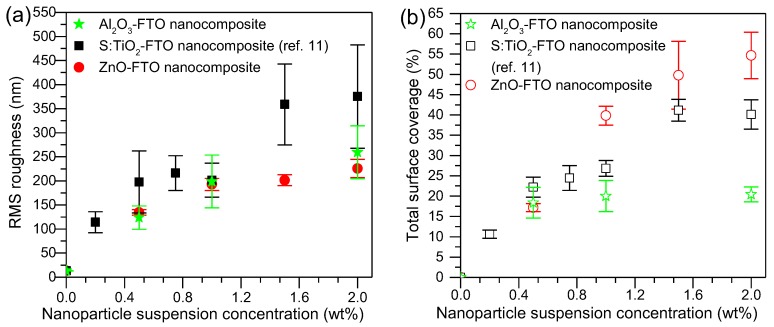
(**a**) Root-mean-square (RMS) roughness and (**b**) total surface coverage plotted against nanoparticle suspension concentration for Al_2_O_3_-FTO, S:TiO_2_-FTO (as reported in [11]), and ZnO-FTO nanocomposites. At least five different areas were measured on each sample to deduce the corresponding values and associated error bars.

Figure 2a shows the root-mean-square (RMS) roughness of Al_2_O_3_-FTO nanocomposites, which was calculated from AFM images (40 × 40 μm^2^ with resolution of 40 nm per pixel) in the same manner as in [11]. The roughness of S:TiO_2_-FTO nanocomposites (as in [11]) and ZnO-FTO (recalculated in the same way as in this study to enable a fair comparison, and thus different from that of [10] are also plotted for comparison. With increasing nanoparticle suspension concentration, the RMS roughness of Al_2_O_3_-FTO nanocomposites increases, being comparable with that of ZnO-FTO nanocomposites and lower than that of S:TiO_2_-FTO nanocomposites.

**Figure 3:**
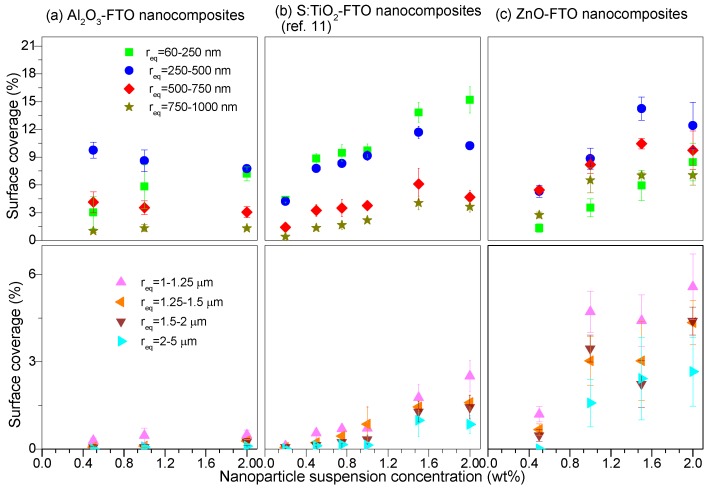
Surface coverages calculated for all eight groups (classified based on the equivalent radius r_eq_) of nanoparticle agglomerates for (**a**) Al_2_O_3_-FTO nanocomposites; (**b**) S:TiO_2_-FTO nanocomposites (as reported in [11]); and (**c**) ZnO-FTO nanocomposites. For each series, the top and bottom panels show the surface coverage of groups with r_eq_ inferior and superior to 1 μm, respectively.

In addition, another important parameter that can be obtained from AFM images is the total surface coverage, defined as the percentage of the area occupied by all nanoparticle agglomerates divided by the total image area. As seen in Figure 2b, with increasing nanoparticle suspension concentration, the total surface coverage of Al_2_O_3_-FTO reaches a plateau near 20%, as opposed to what was observed for ZnO-FTO and S:TiO_2_-FTO nanocomposites, for which total surface coverage increases constantly with nanoparticle suspension concentration. In order to get an in-depth understanding of surface morphologies characteristic of Al_2_O_3_-FTO nanocomposites, the size distribution of the nanoparticle agglomerates was statistically analyzed and compared to that of ZnO-FTO and S:TiO_2_-FTO nanocomposites in Figure 3. The nanoparticle agglomerates are divided into eight groups based on their equivalent radius r_eq_ (defined as the effective radius of a circle whose area is equivalent to the projected area of the nanoparticle agglomerate): 60–250 nm, 250–500 nm, 500–750 nm, 750–1000 nm, 1–1.25 μm, 1.25–1.5 μm, 1.5–2 μm, and 2–5 μm. The surface coverage associated to each group can then be deduced as plotted in Figure 3.

For the sake of clarity, the latter four groups corresponding to larger nanoparticle agglomerates (r_eq_ > 1 μm) are drawn separately from the former four groups (smaller nanoparticle agglomerates with r_eq_ < 1 μm). It should be noted that we have not taken into account the nanoparticle agglomerates with r_eq_ < 60 nm as they are out of the resolution of the AFM images analyzed here. Generally speaking, all three series of FTO nanocomposites tend to form nanoparticle agglomerates with r_eq_ less than 1 μm. However, S:TiO_2_-FTO, and particularly ZnO-FTO nanocomposites also show a fairly high proportion of agglomerates with r_eq_ > 1 μm, the surface coverages of which significantly increase with increasing nanoparticle suspension concentration. For a target application in solar cells, it is well known that large feature sizes can generate technical problems for cell processing [19,20] (since it can for instance induce a local shunt). In comparison, the portion of large-sized nanoparticle agglomerates in Al_2_O_3_-FTO nanocomposites is almost negligible. Further, in Al_2_O_3_-FTO nanocomposites about 90% of agglomerates show r_eq_ < 750 nm while about 80% in ZnO-FTO and S:TiO_2_-FTO nanocomposites show r_eq_ < 1.25 μm and r_eq_ < 1 μm, respectively. In this regard, among the three series of nanocomposites, Al_2_O_3_-FTO nanocomposites appear most favorable to be integrated in solar devices that are highly sensitive to TCO roughness and large feature sizes.

### 3.2. Structural and Electrical Properties of Al_2_O_3_-FTO Nanocomposites

**Figure 4:**
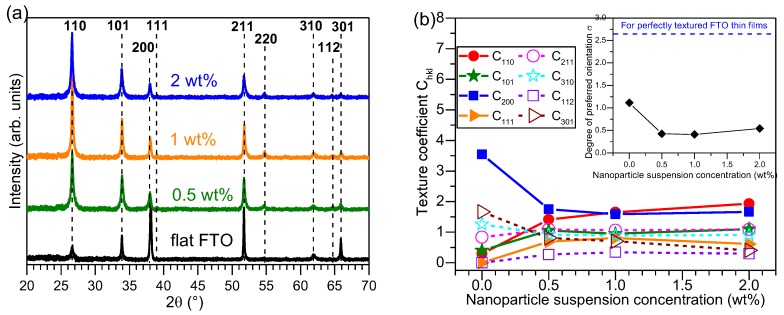
(**a**) X-ray diffraction (XRD) θ-2θ patterns of Al_2_O_3_ nanocomposites and respective reference flat FTO. The dashed lines mark the diffraction peaks of FTO (PDF: SnO_2_ 00-041-1445); (**b**) Texture coefficient C_hkl_ plotted against nanoparticle suspension concentration for Al_2_O_3_-FTO nanocomposites. The inset shows the evolution of the degree of preferred orientation σ.

The X-ray diffraction (XRD) patterns of Al_2_O_3_-FTO nanocomposites are presented in Figure 4a where the diffraction peaks corresponding to FTO are marked with dashed lines. No visible reflection corresponding to Al_2_O_3_ nanoparticles were observed, indicating the amorphous nature of the Al_2_O_3_ nanoparticles. The texture coefficient C_hkl_ for (110), (101), (200), (111), (211), (310), (112), and (301) planes of FTO are calculated according to Harris’s method [21]:

(1) $$C_{hkl}=\;\frac{\frac{I_{hkl}}{I_{0,\;hkl}}}{\frac{1}{N}\;\sum_{N}\frac{I_{hkl}}{I_{0,\;hkl}}}$$

where I_hkl_ and I_0,hkl_ represent the experimental and reference (taken from the powder diffraction files) diffraction intensity, respectively; while N equals the total number of diffraction peaks. The C_hkl_ for each plane is plotted as a function of nanoparticle suspension concentration in Figure 4b, where the inset shows the evolution of the degree of preferred orientation σ obtained as:

(2) $$\sigma \;=\frac{\sqrt{\sum_{N}{{(C}_{hkl}-1)}^{2}}}{\sqrt{N}}$$

When growing on top of Al_2_O_3_ nanoparticles, FTO grains do not align in the same orientations as those grown on bare glass surface. Thus, these FTO grains expose different crystal planes to the incident X-rays compared to the reference flat FTO. Consequently, as one sees in Figure 4a,b, the texture of Al_2_O_3_-FTO nanocomposites is different from that of reference flat FTO. Furthermore, due to the presence of Al_2_O_3_ nanoparticles, the FTO grains are more randomly aligned. In other words, Al_2_O_3_-FTO nanocomposites structurally behave more like powder samples with random orientations. So the C_hkl_ of all diffraction peaks for Al_2_O_3_-FTO nanocomposites evolve towards 1 and also the degree of preferred orientation drops. The structural texture of Al_2_O_3_-FTO nanocomposites remains almost constant with varying nanoparticle suspension concentration. Unlike the local epitaxy observed between S:TiO_2_ nanoparticles and FTO in [11], the Al_2_O_3_ nanoparticles does not seem to impose any restrictions on the growth of subsequent FTO thin films, resulting in a more random orientation of the FTO grains.

**Figure 5:**
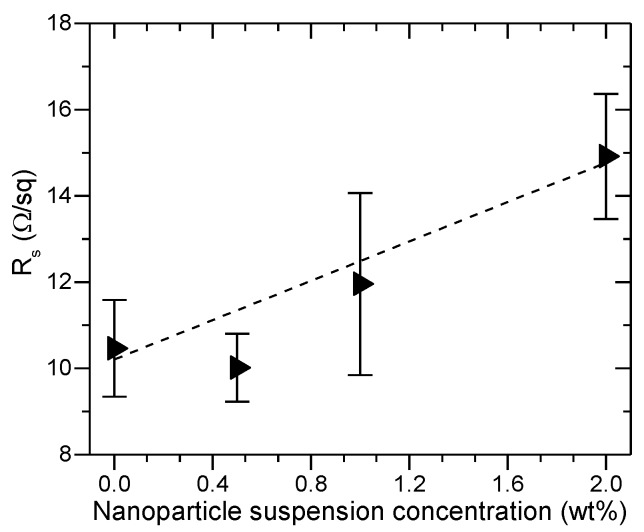
Sheet resistance (R_s_) with respect to nanoparticle suspension concentration plotted for Al_2_O_3_-FTO nanocomposites. The error bar of each specimen was obtained from statistical analysis on measurements at five different regions. The dashed line is a guide to the eye.

The geometrical randomness imposed by the presence of nanoparticles also results in an increase of sheet resistance (R_s_) in Al_2_O_3_-FTO nanocomposites, as seen in Figure 5. To a first approximation, if one considers solely the FTO film in the nanocomposites, its cross section can be assumed constant (since the FTO film is conformally coated on top of nanoparticle agglomerates). Therefore, one expects the total volume of FTO film to be larger in nanocomposites than in the reference flat FTO. That is to say, according to the Pouillet’s law, the electrical carriers in FTO nanocomposites should travel a longer effective length: the higher the nanoparticle suspension concentration, the rougher the nanocomposite and the longer the effective length. Unlike the S:TiO_2_ nanoparticles reported in [11], the Al_2_O_3_ nanoparticles used in this work are not doped and are thus electrically insulating. When an electrical current is injected into Al_2_O_3_-FTO nanocomposites, the current is expected to flow only through the FTO film, encountering a larger resistance due to increased length. The large roughness is also expected to increase electrical recombination losses at the surface/interfaces, thus contributing to the increased resistance. Consequently, R_s_ of Al_2_O_3_-FTO nanocomposite is larger than that of reference flat FTO and increases further with increasing nanoparticle suspension concentration. The largest increase in R_s_ is observed in the 2 wt % Al_2_O_3_-FTO nanocomposite by 42%, which is nevertheless smaller than the increase of 57% observed in ZnO-FTO nanocomposite in [10]. It should be mentioned that despite the relative increase with respect to the reference flat FTO, R_s_ of Al_2_O_3_-FTO still falls into the suitable range for PV applications [22].

### 3.3. Optical Properties of Al_2_O_3_-FTO Nanocomposites

**Figure 6:**
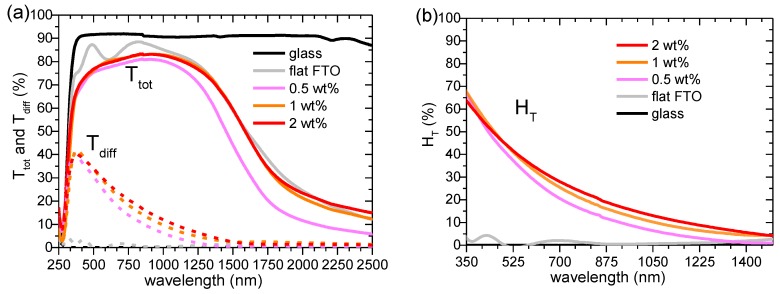
(**a**) Total transmittance (T_tot_) and diffuse transmittance (T_diff_) for Al_2_O_3_-FTO nanocomposites and respective reference flat FTO as well as the bare glass substrate; (**b**) Haze factor (H_T_) for Al_2_O_3_-FTO nanocomposites and respective reference flat FTO as well as the bare glass substrate.

The total (T_tot_) and diffuse (T_diff_) transmittance in 250–2500 nm range for Al_2_O_3_-FTO nanocomposites is shown in Figure 6a and the respective haze factor in transmittance (H_T_) in 350–1500 nm is shown in Figure 6b. The haze factor in transmittance H_T_ is defined as:

(3) $$H_{T}=\frac{T_{diff}}{T_{tot}}$$

where T_tot_ equals to the sum of the specular transmittance (T_spec_) and T_diff_. Note that H_T_, T_tot_, and T_diff_ are wavelength dependent.

**Figure 7:**
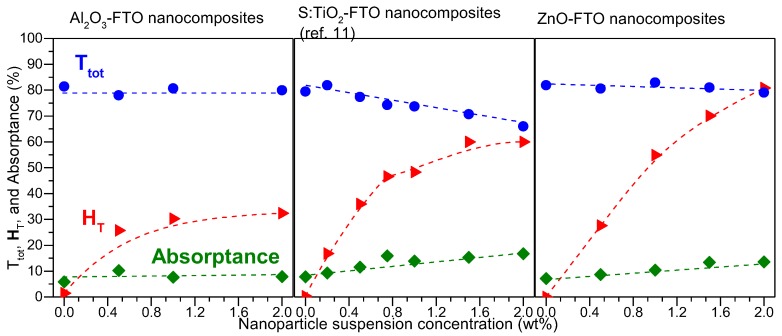
Haze factor (H_T_), total transmittance (T_tot_), and absorptance at 635 nm for Al_2_O_3_-FTO, S:TiO_2_-FTO (as reported in [11]), and ZnO-FTO nanocomposites plotted as a function of nanoparticle suspension concentration. Dashed lines help to guide the eyes.

The reference flat FTO, as expected, shows essentially zero H_T_ at all wavelengths. Upon increasing the nanoparticle suspension concentration, H_T_ of Al_2_O_3_-FTO nanocomposites appears to increase only slightly, as opposed to the drastic change observed in ZnO-FTO (reported in [10]) and S:TiO_2_-FTO nanocomposites (reported in [11]. To render a detailed comparison, Figure 7 plots T_tot_, H_T_, and absorptance for Al_2_O_3_-FTO, S:TiO_2_-FTO, and ZnO-FTO nanocomposites as a function of nanoparticle suspension concentration at a single visible wavelength, 635 nm in our case (since the visible range is of most interest for optoelectronic applications). The slight differences between the respective references flat FTOs for the three series of nanocomposites originate from the batch-to-batch differences. On the one hand, Al_2_O_3_-FTO nanocomposites exhibit a modest H_T_ of 32.3% compared to ZnO-FTO nanocomposite (highest H_T_ of 80.9%) and S:TiO_2_-FTO nanocomposite (highest H_T_ of 60.0%). On the other hand, T_tot_ in Al_2_O_3_-FTO nanocomposite is almost invariant with a negligible loss of 1.8%. Whereas in ZnO-FTO nanocomposites a slightly higher drop in T_tot_ by 3.5% is observed and S:TiO_2_-FTO nanocomposites show the highest loss in T_tot_ (up to 17%). For all three series of nanocomposites, the reduction in T_tot_ is accompanied by a simultaneous increase in absorptance, suggesting that the loss in transmittance is caused by an enhanced absorption. When passing through the FTO film deposited on top of the nanoparticle agglomerates, light essentially experiences a longer path compared to passing through the flat FTO film. Despite their roughness comparable with ZnO-FTO nanocomposites, Al_2_O_3_-FTO nanocomposites have a much smaller portion of large-sized nanoparticle agglomerates and thus experience the least absorption. As for S:TiO_2_-FTO nanocomposites, the higher absorption in S:TiO_2_-FTO is attributed to the additional absorption by S:TiO_2_ nanoparticles due to the defect levels induced by S-doping as reported in [11], and thus T_tot_ experiences the most severe reduction.

For the Al_2_O_3_-FTO and related FTO nanocomposites studied here, one clearly sees that depending on the nanoparticle selected, different tradeoffs between H_T_, T_tot_, and R_s_ would result, which offers a great flexibility to design and fabricate FTO and other TCO nanocomposites with optimized properties orientated for specific target application. For example, despite their medium H_T_ (~30%), Al_2_O_3_-FTO nanocomposites nevertheless present small feature sizes and thus should appear suitable for applications such as (planar) organic solar cells which are highly sensitive to TCO surface roughness [23]; while ZnO-FTO and S:TiO_2_-FTO nanocomposites are better used in applications such as dye-sensitized solar cells (DSSCs) which appear less sensitive to large feature sizes but require high H_T_ of TCO materials.

### 3.4. Modeling of Light Scattering by a Single Nanoparticle Agglomerate

Effectively, for the Al_2_O_3_-FTO and related FTO nanocomposites investigated here, the nanoparticle agglomerates exhibit a broad size distribution: some agglomerates appear to exhibit a size much larger (see Figure 3) than the FTO film thickness (~300 nm). Consequently, it is less proper to consider these FTO nanocomposites as a “homogenous media” as usually considered in literature for other textured TCOs like the Asahi type-U or W-textured FTO [24,25]. Instead, here it is more reasonable to treat these nanoparticle agglomerates as “grains”, each functioning as an individual scattering center. Therefore, the optical scattering of the resultant nanocomposites is related to the overall scattering by all the “grains” present. Thus, understanding the optical scattering of an FTO nanocomposite containing a single “grain” is of key importance. To do so, we propose here a simple optical model in which we treat the “grain” (i.e., nanoparticle agglomerate) as a phase object φ:

(4) $$\varphi =\exp\left[j\cdot \frac{2\pi }{\lambda }\cdot n\cdot z\left(x,y\right)\right]$$

where z(x,y) corresponds to the height of the top surface of the nanoparticle agglomerate in question (z varies as a function of the calculation coordinates x and y); λ refers to the wavelength and n is the refractive index of the nanoparticle. The transmittance can then be calculated as [26]:

(5) $$T=T_{0}\cdot \exp\left[-\;{\left(\alpha_{i}-\alpha_{t}\right)}^{2}\delta^{2}\right]\cdot \varphi$$

Here T_0_ stands for the transmittance of a perfectly flat surface. The influence of the surface roughness δ, including the interface between the nanoparticle agglomerates and FTO, as well as that of FTO and air, is expressed in the exponential term; The parameter of wave factor α is defined as: α/2π = ncosθ/λ (n being the refractive index of respective optical media), which in this case can essentially be simplified to α = 2πn/λ with incidence angle being 0°. The two subscripts “i” and “t” refer to the incident and transmitted light, respectively.

**Figure 8:**
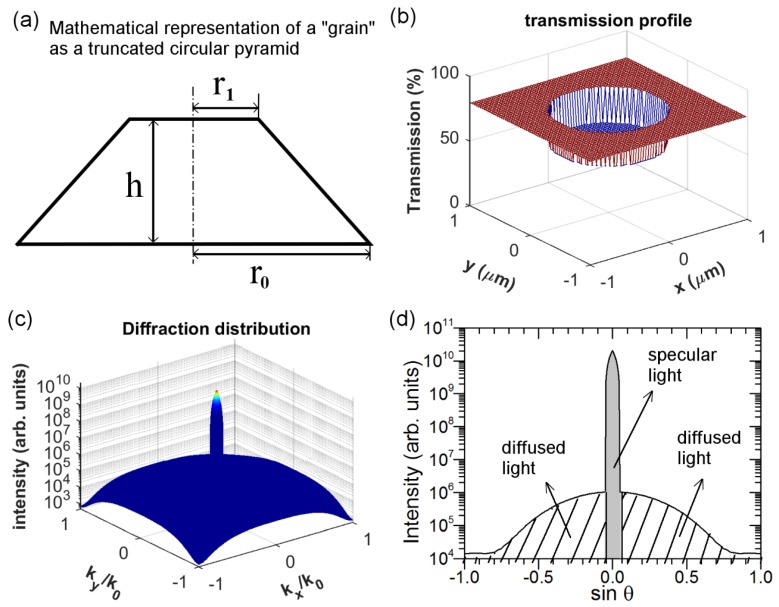
(**a**) Mathematical representation of an individual nanoparticle agglomerate approximated as a truncated circular pyramid described by three parameters: bottom radius r_0_, top radius r_1_, and height h; (**b**) The profile of total transmission for light passing through FTO nanocomposite containing a ZnO nanoparticle agglomerate parameterized as r_0_ = 600 nm, r_1_ = 200 nm, and h = 150 nm; (**c**) 3D-plot of light intensity in k-space; and (**d**) 2D-plot of light intensity, deduced as cross section at k_y_/k_0_ = 0 (or equivalently, k_x_/k_0_ = 0) in (**c**). The specularly (corresponding to about 3°) and diffusely transmitted light are pointed out, respectively.

In a first approximation, the cross section of a single nanoparticle agglomerate (i.e., “grain”) can be mathematically represented by a truncated circular pyramid defined by three variables: the bottom radius r_0_; the top radius r_1_; and finally the height h as in Figure 8a. The cross-section SEM image of a single agglomerate in Figure S2 and height profiles of several grains examined in AFM images in Figure S3 support such assumption. As detailed previously, compared to Al_2_O_3_-FTO and S:TiO_2_-FTO nanocomposites, ZnO-FTO nanocomposites show the widest size distribution of “grains” thus are able to provide sufficient sample “grains” for screening purpose in the model. Therefore, the illustration of the optical model is exemplified with ZnO-FTO nanocomposite in the following discussion. As an example, Figure 8b–d simulates the optical scattering of FTO nanocomposite containing a single ZnO nanoparticle agglomerate at λ = 635 nm. The size of the ZnO nanoparticle agglomerate is randomly selected, in this example we have chosen r_0_, r_1_, and h to be 600 nm, 200 nm, and 150 nm, respectively. In passing through such a ZnO nanoparticle agglomerate, the total transmission can be then calculated from Equation (4) and is plotted in Figure 8b. The transmission, as expected, appears lower in the region where the ZnO nanoparticle agglomerate is located (one can note that the central part sinks in Figure 8b). By performing the Fourier transform of the data reported in Figure 8b [27], one can calculate the transmitted light intensity in k-space, as shown in Figure 8c where both axes correspond to the sine of the diffraction angle (θ), i.e., k_x_/k_0_ = k_y_/k_0_ = sinθ. Figure 8d draws a 2D-plot of the transmitted light intensity versus sinθ to better show the transmitted light for different angles, which essentially corresponds to the cross section of Figure 8c for k_y_/k_0_ = 0 (or equivalently, k_x_/k_0_ = 0). As shown in Figure 8d, the specular light (here in this example corresponding to about 3°) appears much more intense than the diffused light. This means that light is mainly scattered in the specular direction by such a ZnO nanoparticle agglomerate. By performing proper integration, one obtains the light flux/power corresponding to specularly (P_spec_) and totally (P_tot_) transmitted light, whose difference gives the diffusely transmitted light power (P_diff_): P_diff_ = P_tot_ − P_spec_. Consequently, the haze factor of light passing through FTO nanocomposite containing a single nanoparticle agglomerate is: H_T_ = P_diff_/P_tot_.

**Figure 9:**
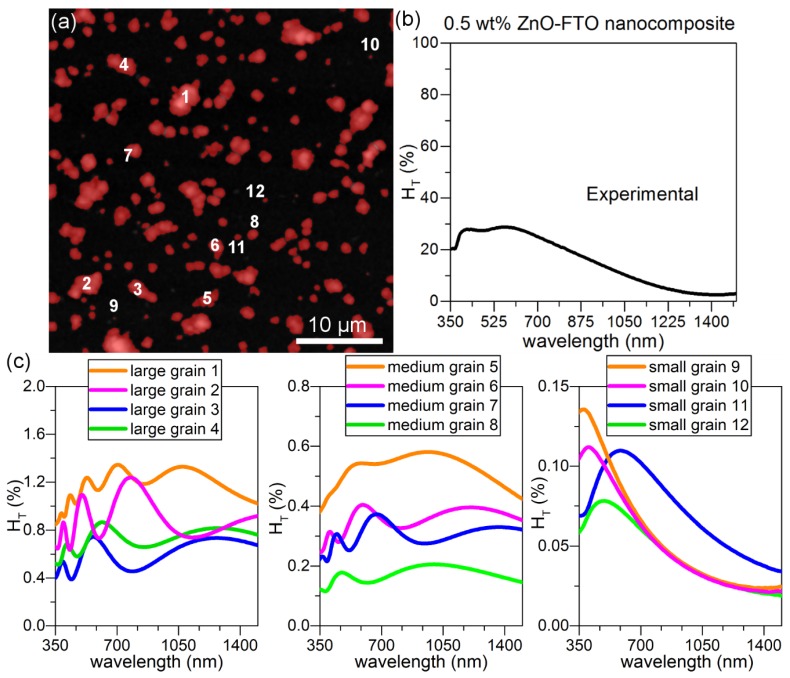
(**a**) AFM image of 40 × 40 μm^2^ for a 0.5 wt % ZnO-FTO nanocomposite. Here the nanoparticle agglomerates are colored in red with the help of Gwyddion software [28]; (**b**) Plot of H_T_(λ) (measured experimentally) for 0.5 wt % ZnO-FTO nanocomposite; (**c**) Simulated H_T_(λ) of FTO nanocomposite containing a single grain of those marked 1–12 in (**a**), respectively. Here the specular light is considered as the central one pixel only.

This model is then used to simulate real individual nanoparticle agglomerates present in a 0.5 wt % ZnO-FTO as reported in Figure 9a where the nanoparticle agglomerates are counted as “grains” and colored in red for the sake of clarity. It is seen that the grain size distribution is rather broad and roughly they can be classified within three groups as shown by the randomly selected twelve grains: large grains 1–4 and medium grains 5–8, as well as small grains 9–12. As a reference, the r_eq_ (as defined previously) of grains 1–5 are superior to 1 μm while that of grains 6–12 are inferior to 1 μm. Detailed r_eq_ values of grain 1–12 are tabulated in Table S1. The H_T_(λ) for each of these 12 grains is simulated in Figure 9c. For simplicity, the central one pixel is considered as the specular light. The large grains (grains 1–4) exhibit the largest H_T_(λ) value; moreover, significant interference fringes can be seen. With decreasing grain size, the fringes gradually disappear (from grains 5–8 to grains 9–12), and the H_T_(λ) is also observed to decrease accordingly (one notes that the scale of H_T_ changes in Figure 9c), which suggests that among all grains the larger grains should have a more significant contribution to scattering light in such type of FTO nanocomposites. In order to simplify the calculation without altering the physical significance, it is most ideal to choose a small grain as grains 9–12 which does not show fringes and also best resembles the shape of H_T_(λ) curve of the real nanocomposite (Figure 9b).

**Figure 10:**
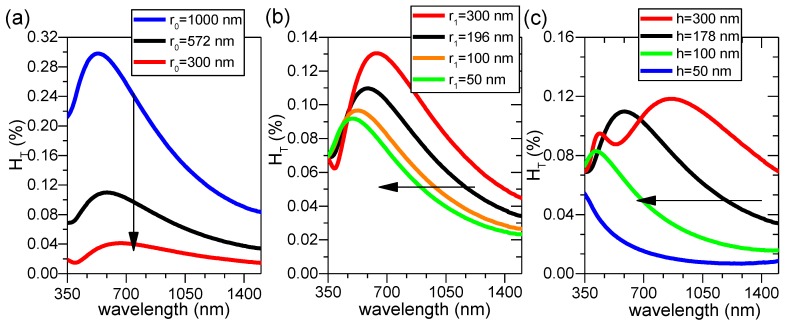
Simulated H_T_(λ) of FTO nanocomposite containing a single ZnO nanoparticle agglomerate: (**a**) r_0_ varies while r_1_ and h are fixed to 196 and 178 nm, respectively; (**b**) r_1_ varies while r_0_ and h are fixed to 572 and 178 nm, respectively; and (**c**) h varies while r_0_ and r_1_ are fixed to 572 and 196 nm, respectively. The specular light is considered as the central one pixel only.

Hence, a grain with r_0_, r_1_, and h of 572, 196, and 178 nm (same as that of grain 11) is exemplified in Figure 10 in order to study the individual influence of bottom and top radius (r_0_ and r_1_), as well as height (h), on H_T_(λ). In Figure 10a, with fixed r_1_ and h values, when r_0_ is decreased, H_T_(λ) value shifts down to lower amplitude while the same shape remains. In Figure 10b, with fixed r_0_ and h, when r_1_ is decreased, H_T_(λ) maintains more or less the same shape but shifts to the left with the amplitude slightly lowered. Finally if r_0_ and r_1_ are fixed as in Figure 10c, with decreasing h, the shape of H_T_(λ) appears to change relatively significantly. This is effectively due to the extreme sensitivity of optical fringes on height. As is well known for thin film materials, the slight change in film thickness would immediately result in the change of optical fringes; consequently, the wavelengths corresponding to peaks/valleys of fringes also change.

**Figure 11:**
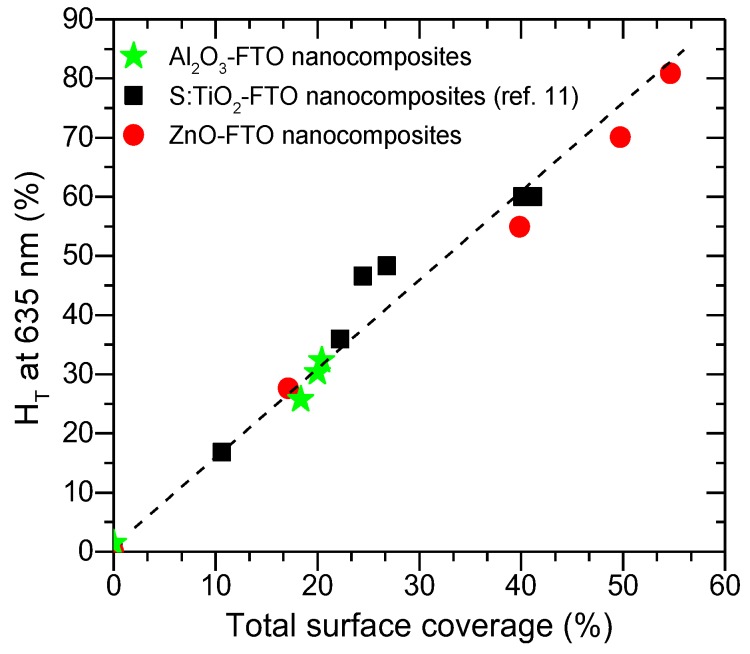
Haze factor (H_T_) at 635 nm as a function of the total surface coverage for Al_2_O_3_-FTO, S:TiO_2_-FTO (reported in [11]), and ZnO-FTO nanocomposites.

Of the three parameters examined here, one concludes that the bottom radius r_0_ affects the optical scattering most while the top radius r_1_ shows least influence and height h affects the optical scattering by affecting the appearance of optical fringes. As the bottom radius r_0_ is essentially related to the projected area of the nanoparticle agglomerates, one would expect a direct correlation between the total surface coverage and the optical scattering for such type of nanocomposites. As is seen in Figure 11, when drawing H_T_ at 635 nm against the total surface coverage for Al_2_O_3_-FTO, S:TiO_2_-FTO and ZnO-FTO nanocomposites, a fairly straight line is observed, which is consistent with the simulated results. When varying the nanoparticle suspension concentration, the total surface coverage of Al_2_O_3_-FTO nanocomposites remains relatively constant, and consequently their H_T_(λ) remains almost invariant. Further work is ongoing to develop a more complex model to simulate the H_T_ of final nanocomposites, which would require more sophisticated physical considerations such as the interaction among nanoparticle agglomerates, and/or the free carrier absorption within the FTO film in the near-infrared range, etc.

## 4. Conclusions

In this work, we have successfully developed newly hazy Al_2_O_3_-FTO nanocomposites by combining Al_2_O_3_ nanoparticle with F:SnO_2_ (FTO) thin films. The presence of the Al_2_O_3_ nanoparticles induces the FTO grains to grow with more random orientations as is confirmed by XRD data, and as opposed to the local epitaxial growth of FTO grains on rutile S:TiO_2_ nanoparticle agglomerates [11]. In terms of electrical properties, while Al_2_O_3_-FTO nanocomposites show an increase of sheet resistance R_s_ by up to 42%, the absolute R_s_ values obtained still fall into the suitable range for photovoltaics applications. Compared to ZnO-FTO and S:TiO_2_-FTO nanocomposites reported previously, Al_2_O_3_-FTO nanocomposites show medium haze factor (H_T_) of about 30% (as opposed to 80% and 60% for ZnO-FTO and S:TiO_2_-FTO nanocomposites, respectively) but they maintain the least loss in total transmittance (T_tot_*)* by 1.8% (as opposed to 3.5% and 17% for ZnO-FTO and S:TiO_2_-FTO nanocomposites, respectively); most importantly, they show the lowest portion of large-sized nanoparticle agglomerates and smaller feature sizes (90% of the agglomerates exhibiting equivalent radius r_eq_ less than 750 nm). Therefore, Al_2_O_3_-FTO nanocomposites appear promising to be used in applications that are highly sensitive to rough TCO surface and large feature sizes.

We have also presented a simple optical model to simulate the optical scattering of FTO nanocomposite containing a single nanoparticle agglomerate defined by three parameters: bottom radius r_0_, top radius r_1_, and height h. By modeling separately the influence of r_0_, r_1_, and h on the H_T_(λ), the bottom radius r_0_ is found to affect most significantly the optical scattering. This demonstrates that the H_T_ of such type of FTO nanocomposite (i.e., Al_2_O_3_-FTO and related FTO nanocomposites) is essentially determined by the total surface coverage (of all nanoparticle agglomerates), which is consistent with the experimental observations.

Our results provide important complements in designing hazy FTO and other TCO nanocomposites with optimized functional properties targeted for various photovoltaics and optoelectronics applications.

## Supplementary Materials

The following are available online at http://www.mdpi.com/2079-4991/8/6/440/s1, Figure S1: Schematic drawing of the two-step process (not to scale) for fabricating Al_2_O_3_-FTO nanocomposites, Figure S2: SEM image of a 1 wt % S:TiO_2_-FTO nanocomposite presenting the cross section of a nanoparticle agglomerate, which resembles and thus is approximated as a truncated circular pyramid, Figure S3: (a) AFM image of a 1 wt % ZnO-FTO nanocomposite; right panel summarizes the height profiles of the six grains indicated; (b) AFM image of a 1wt % S:TiO_2_-FTO nanocomposite; right panel summarizes the height profiles of the six grains indicated; (c) AFM image of a 1 wt% Al_2_O_3_-FTO nanocomposite; right panel summarizes the height profiles of the six grains indicated, Table S1: The values of equivalent radius r_eq_ of the grains 1–12 marked in Figure 9 in the main text.
