# Supplementary material for: Hazy Al2O3-FTO Nanocomposites: A Comparative Study with FTO-Based Nanocomposites Integrating ZnO and S:TiO2 Nanostructures
Source: Nanomaterials (Basel). 2018 Jun 16;8(6):440. doi: 10.3390/nano8060440 (PMC6027414; doi:10.3390/nano8060440)
Supplement: Supplementary file 1 [file nanomaterials-08-00440-s001.pdf]

Supporting Information

Hazy Al_2O_3-FTO Nanocomposites: A Comparative Study with FTO-Based Nanocomposites Integrating ZnO and S:TiO_2 Nanostructures

Shan-Ting Zhang [1,2,*], Guy Vitrant [3], Etienne Pernot [1], Carmen Jiménez [1], David Muñoz-Rojas [1] and Daniel Bellet [1,*]

1 Université Grenoble Alpes, CNRS, Grenoble INP, LMGP, F-38000 Grenoble, France; etienne.pernot@grenoble-inp.fr (E.P.); carmen.jimenez@grenoble-inp.fr (C.J.); David.Munoz-Rojas@grenoble-inp.fr (D.M.-R.)
2 Technische Universität Darmstadt, Jovanka-Bontschits-Strasse 2, Darmstadt 64287, Germany
3 Université Grenoble Alpes, CNRS, Grenoble INP, IMEP-LAHC, F-38000 Grenoble, France; guy.vitrant@minatec.grenoble-inp.fr
* Correspondence: shanting.zhang@gmail.com (S.-T.Z.); daniel.bellet@grenoble-inp.fr (D.B.); Tel.: +33-4-56-52-93-37 (S.-T.Z. & D.B.)

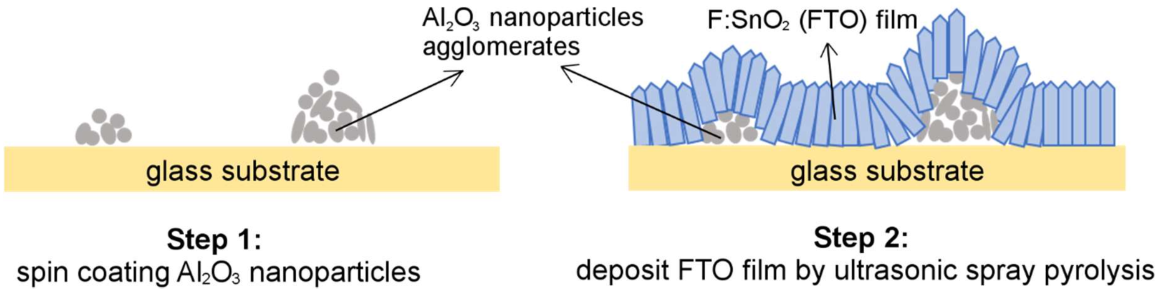

Figure S1: Schematic drawing of the two-step process (not to scale) for fabricating Al_2O_3-FTO nanocomposites.

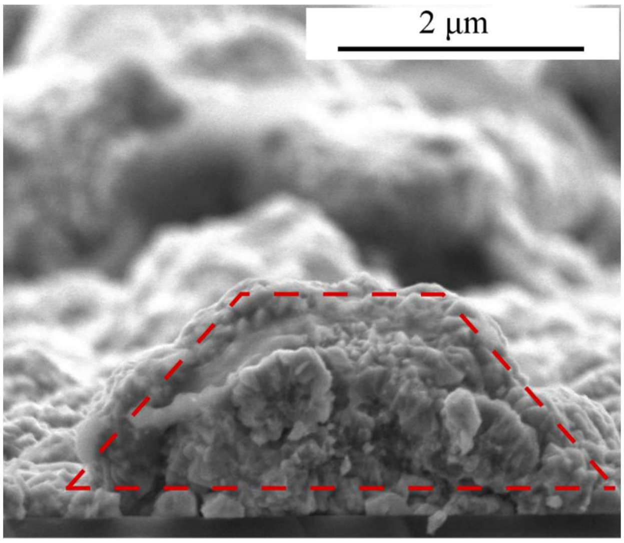

Figure S2: SEM image of a 1 wt % S:TiO_2-FTO nanocomposite presenting the cross section of a nanoparticle agglomerate, which resembles and thus is approximated as a truncated circular pyramid.

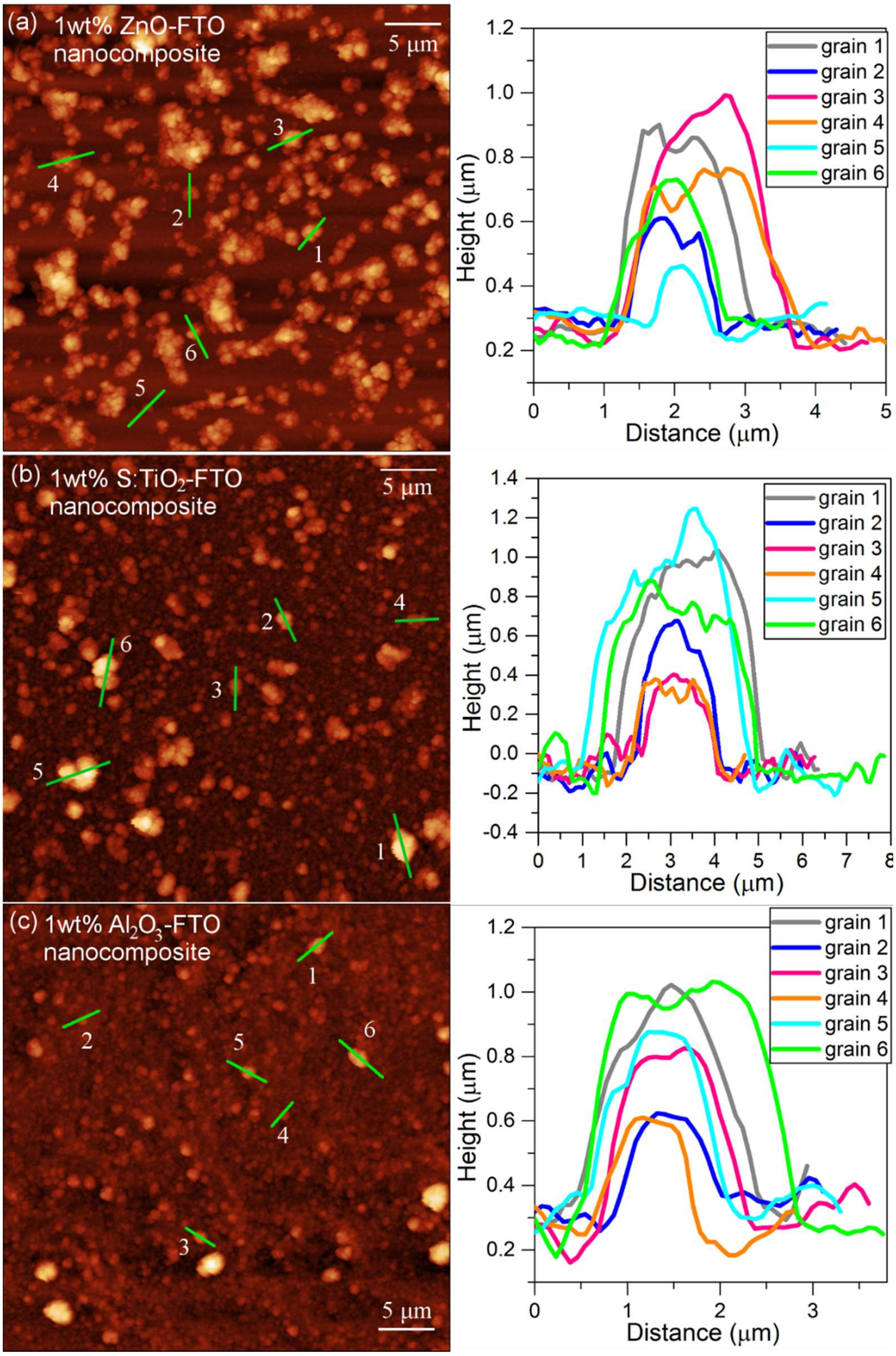

Figure S3: (**a**) AFM image of a 1 wt % ZnO-FTO nanocomposite; right panel summarizes the height profiles of the six grains indicated; (**b**) AFM image of a 1 wt % S:TiO_2-FTO nanocomposite; right panel summarizes the height profiles of the six grains indicated; (**c**) AFM image of a 1 wt % Al_2O_3-FTO nanocomposite; right panel summarizes the height profiles of the six grains indicated.

Table S1: The values of equivalent radius r_{eq} of the grains 1–12 marked in Figure 9 in the main text.

	**r_{eq} (nm)**		**r_{eq} (nm)**		**r_{eq} (nm)**
grain 1	1800	grain 5	1060	grain 9	98.6
grain 2	1590	grain 6	919.5	grain 10	170.75
grain 3	1220	grain 7	881.7	grain 11	360.9
grain 4	1340	grain 8	610.9	grain 12	253.2
